# The Natural Antimicrobial Enzyme Lysozyme is Up-Regulated in Gastrointestinal Inflammatory Conditions

**DOI:** 10.3390/pathogens3010073

**Published:** 2014-01-16

**Authors:** Carlos A. Rubio

**Affiliations:** Gastrointestinal and Liver Pathology Research Laboratory, Department of Pathology, Karolinska Institute and University Hospital, Stockholm 17176, Sweden; E-Mail: Carlos.Rubio@ki.se; Tel.: +46-8-51774527; Fax: +46-8-51774524

**Keywords:** lysozyme, chronic inflammation, oesophagus, stomach, duodenum, colon

## Abstract

The cells that line the mucosa of the human gastrointestinal tract (GI, that is, oral cavity, oesophagus, stomach, small intestine, large intestine, and rectum) are constantly challenged by adverse micro-environmental factors, such as different pH, enzymes, and bacterial flora. With exception of the oral cavity, these microenvironments also contain remnant cocktails of secreted enzymes and bacteria from upper organs along the tract. The density of the GI bacteria varies, from 10^3^/mL near the gastric outlet, to 10^10^/mL at the ileocecal valve, to 10^11^ to 10^12^/mL in the colon. The total microbial population (*ca.* 10^14^) exceeds the total number of cells in the tract. It is, therefore, remarkable that despite the *prima facie* inauspicious mixture of harmful secretions and bacteria, the normal GI mucosa retains a healthy state of cell renewal. To counteract the hostile microenvironment, the GI epithelia react by speeding cell exfoliation (the GI mucosa has a turnover time of two to three days), by increasing peristalsis, by eliminating bacteria through secretion of plasma cell-immunoglobulins and by increasing production of natural antibacterial compounds, such as defensin-5 and lysozyme. Only recently, lysozyme was found up-regulated in Barrett’s oesophagitis, chronic gastritis, gluten-induced atrophic duodenitis (coeliac disease), collagenous colitis, lymphocytic colitis, and Crohn’s colitis. This up-regulation is a response directed to the special types of bacteria recently detected in these diseases. The aim of lysozyme up-regulation is to protect individual mucosal segments to chronic inflammation. The molecular mechanisms connected to the crosstalk between the intraluminal bacterial flora and the production of lysozyme released by the GI mucosae, are discussed. Bacterial resistance continues to exhaust our supply of commercial antibiotics. The potential use of lysozyme to treat infectious diseases is receiving much attention.

## 1. Introduction

After birth, the cells that line the mucosa of the human gastrointestinal (GI) tract are increasingly threatened by adverse micro-environmental conditions, such as digestive juices of different pH, a wide variety of active enzymes, and enormous amounts of bacteria. Furthermore, cocktails of bacteria and secretions from upper organs challenge the epithelia of downstream organs along the tract.

### 1.1. Estimated Bacterial Flora in the GI Tract

The density of the bacterial flora in the GI tract is huge; it varies from 10^3^/mL near the gastric outlet, to 10^10^/mL at the ileo-cecal valve, to 10^11^ to 10^12^/mL in the colon. The total microbial population (*ca.* 10^14^) [1] exceeds the total number of cells in the GI tract. About 500 to 1,000 different species exist, a biomass that weights about 1.5 kg [1]. Calculating an average genome size for 1,000 *Escherichia coli* species, the number of genes in this microbiome exceeds the total number of human genes by a factor of 100. These bacteria procreate in a luminal bolus that transports a concoction of secretions and mucus from various organs and in the glycocalix extracellular glycoproteins [2]. It is therefore remarkable that despite the *prima facie* inauspicious mixture of harmful secretions and bacteria, the normal GI mucosa retains a healthy state of cell renewal. To counteract the hostile microenvironment, the GI epithelia react by speeding cell exfoliation (the GI mucosa has a turnover time of two to three days), by increasing peristalsis, by eliminating bacteria through secretion of plasma cell-immunoglubulins and by increasing production of natural antibacterial compounds, such as defensin-5 and lysozyme.

### 1.2. The Discovery of Lysozyme

During a deliberate search for medical antibiotics, Alexander Fleming [3] discovered lysozyme, one of the natural defense substances against infection. Lysozyme, also known as muramidase or *N*-acetylmuramide glycanhydrolase, is a family of enzymes (EC 3.2.1.17), which damage bacterial cell walls by catalyzing hydrolysis of 1,4-beta-linkages between *N*-acetylmuramic acid and *N*-acetyl-d-glucosamine residues in a peptidoglycan, and between *N*-acetyl-d-glucosamine residues in chitodextrins [4]. Lysozyme, encoded by the LYZ gene [5], is an ancient enzyme whose origin goes back an estimated 400 to 600 million years [6]. Linkage studies indicate that the lysozyme M and P genes are present on the same chromosome; calculation from both partial protein sequence and phylogenetic data indicate that the duplication that gave rise to those genes occurred about 50 million years ago [5]. Lysozyme is only a generic name (*v. gr.* lysozyme c is a superfamily composed of 88 distinct lysozymes).

### 1.3. Lysozyme Is Up-Regulated in the GI Tract with Inflammation

In a series of immuno-histochemical studies lysozyme was found up-regulated in many organs of the GI undergoing chronic inflammation, such as Barrett’s oesophagitis, chronic gastritis, gluten-induced atrophic duodenitis (coeliac disease), collagenous colitis, lymphocytic colitis, ulcerative colitis (UC), and Crohn’s colitis [7,8,9,10], strongly suggesting that the associated bacterial flora plays an important role in the expression of this antimicrobial enzyme.

## 2. Barrett’s Oesophagus

Following protracted gastric reflux the normal squamous-cell epithelium of the distal oesophagus may undergo columnar-lined (metaplastic) transformation both in humans [11] and in non-human primates [12,13]. The metaplastic transformation in Barrett’s oesophagus includes accessory glands of oxyntic type and/or pyloric type with, or without, intercalated goblet cells [14]. The phenotype carrying goblet cells (GC), known as specialized epithelium or intestinal metaplasia (IM) is regarded, by the American College of Gastroenterology [15], a prerequisite for the histological diagnosis of Barrett’s esophagus (BE). More recently, The British Society of Gastroenterology (BSG) [16] defined Barrett’s oesophagus as a columnar lined oesophagus on biopsies taken from endoscopical areas suggestive of Barrett’s oesophagus. Thus, the presence of GC is not a *sine qua non* requirement for the diagnosis of Barrett’s oesophagus. This new definition by the BSG [16] has gained acceptance both in the rest of Europe [17] and in Asia [18]. Patients with gastro-oesophageal reflux often receive proton pump inhibitor medication [19]. The reduction of gastric acid secretion by this medication encourages bacterial growth in Barrett’s oesophagus and, hence, increased production of nitrosamines with secondary epithelial damage. In fact, oesophageal biopsies with of Barrett´s mucosa often show signs of on-going or past mucosal inflammation [20].

### 2.1. Bacteria in Barrett’s Oesophagus

Several workers demonstrated that special bacteria are more often present in oesophageal biopsies with Barrett’s oesophagus than in those without Barrett’s oesophagus [21,22,23]. Oesophageal microbiomes have been classified into two types: type I is dominated by the genus Streptococcus, concentrated in the phenotypically normal oesophagus, and type II contains a greater proportion of Gram-negative anaerobes/microaerophiles that are primarily found in oesophagitis and Barrett’s oesophagus [24]. In gastro-oesophageal reflux the residential bacterial populations contain 21 distinct species, including *Bacteroidetes*, *Firmicutes*, *Proteobacteria*, and *Actinobacteria* [21]*.* In a more recent study with oesophageal biopsies and aspirates, McFarlane *et al*. [22] found in the mucosa of the Barrett’s oesophagus 46 bacterial species, belonging to 16 genera, with unique levels of *Campylobacter consisus* and *C. rectus*. Taken together, these microbiological findings denote a close association between the occurrence of columnar-lined oesophageal mucosa and the proliferation of abnormal bacteria in the oesophageal microenvironment. More recently, Liu *et al*. found *Firmicutes* (55%), *Proteobacteria* (20%), *Bacteroidetes* (14%), *Fusobacteria* (9%), and *Actinobacteria* (2%), in analysis of 138 16S rDNA sequences from 240 clones of six cases of Barrett’s oesophagus [23].

### 2.2. Lysozyme Is Up-Regulated in Barrett’s Oesophagitis

**Figure 1:**
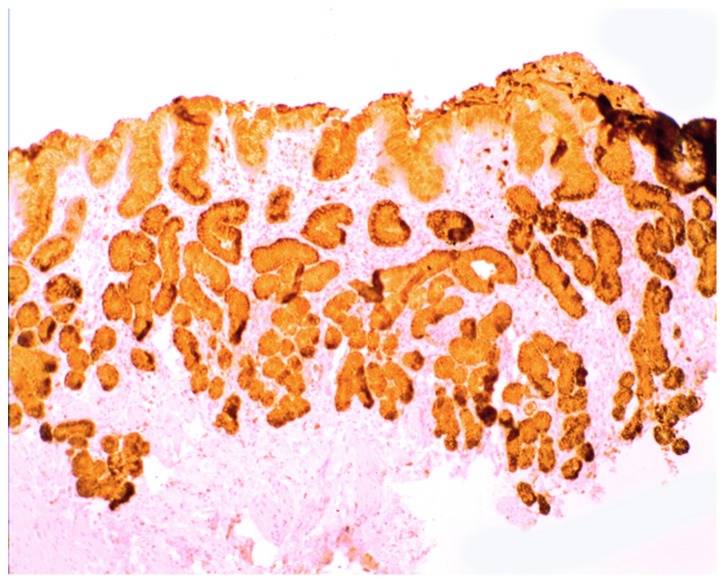
Barrett’s mucosa (pyloric phenotype) showing lysozyme expression in surface epithelium, foveolar epithelium, and pyloric glands (lysozyme immunostain, ×4).

**Figure 2:**
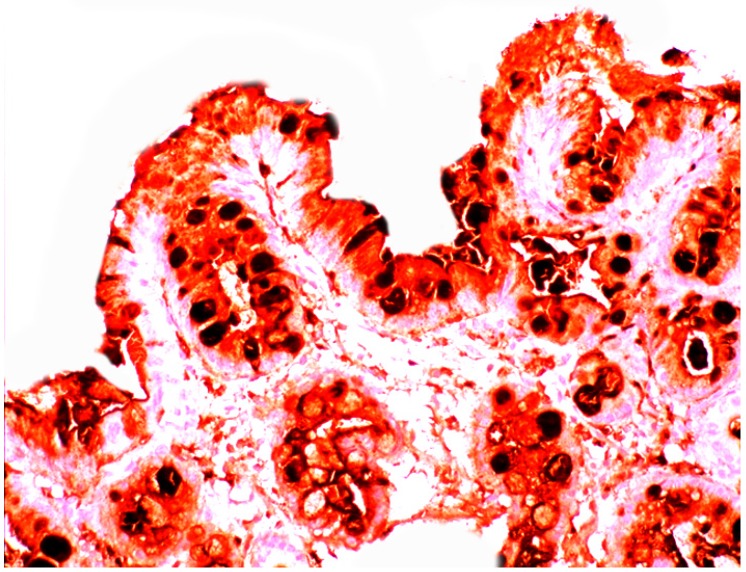
Barrett’s mucosa (intestinal phenotype) showing lysozyme up-regulation, particularly in the luminal epithelial border and in goblet cells (lysozyme immunostain, ×40).

Increased lysozyme immunoreactivity was found In Barrett’s oesophagus; in the surface columnar epithelium, in the columnar epithelium of the pits of the glands (Figure 1), in goblet cells (Figure 2), as well as in Paneth cells in cases with intestinal metaplasia [24].

Intestinal metaplasia in Barrett’s oesophagus is not a mucosal transformation by intestinal cells, but a phenomenon of reconstruction by migrant stem cells of bone marrow origin that adapts to the hostile microenvironment [24]. Circulating bone-marrow cells would engraft into ulcerated mucosal areas affected by on-going chronic inflammation. In a murine model multi-potential progenitor cells of bone marrow origin were found to contribute to the intestinal metaplastic epithelium in the oesophagus [25]. Stem cells in the oesophagus might be instrumental in the molecular cross talk between intraluminal bacterial flora and the production of lysozyme [26]; signals released from the particular bacterial flora might induce stem cells in the Barrett’s oesophagus to generate differentiated cells rich in the antimicrobial enzyme lysozyme.

**Figure 3:**
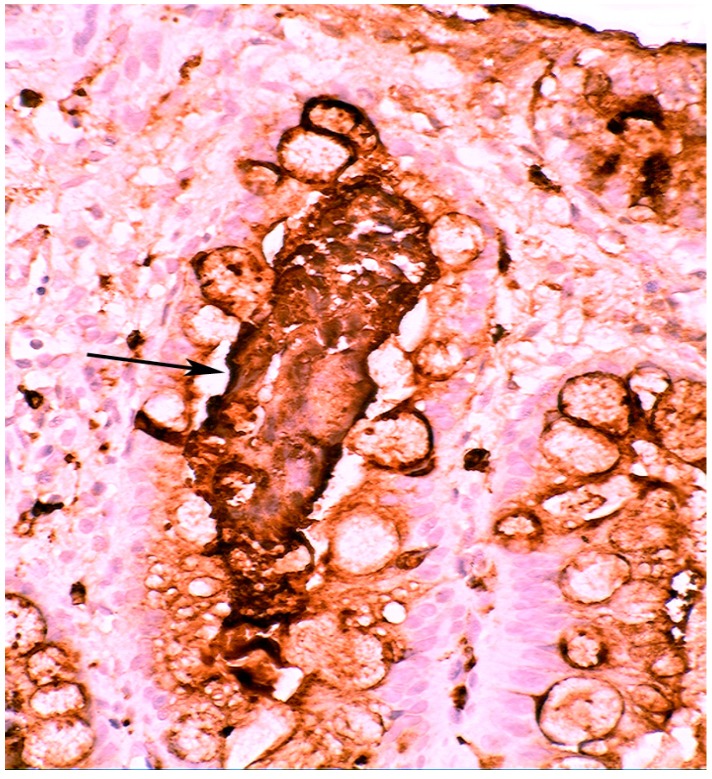
Close-up view of Barrett’s mucosa of intestinal phenotype, showing faintly stained goblet cells and at arrow, marked lysozyme expression in the secreted mucus, in the lumen of the gland (lysozyme immunostain, ×40).

**Figure 4:**
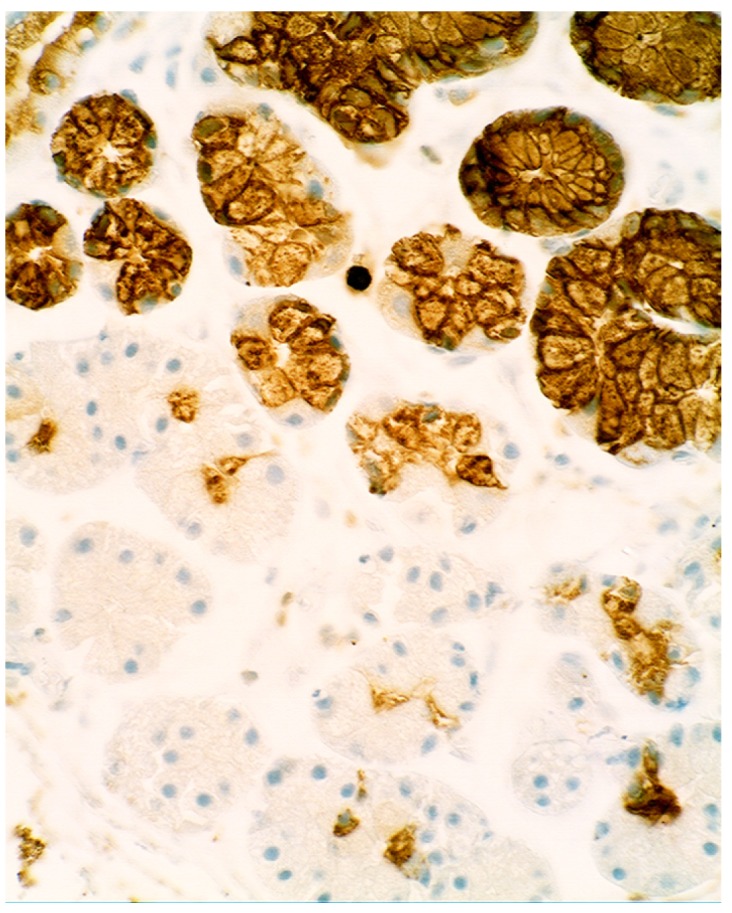
Barrett’s mucosa (oxyntic phenotype), showing lysozyme expression in neck glands. Note negative expression in oxyntic glands (×10).

When compared to controls, lysozyme was up-regulated in all three Barrett’s mucosal phenotypes [24]. In some goblet cells, lysozyme was slightly expressed. This phenomenon might be due to a prior goblet cells-discharge of lysozyme-rich intracellular mucus into the lumen (Figure 3). Lysozyme was not expressed in parietal (oxyntic) cells, neither in Barrett’s oesophagus (Figure 4), nor in controls [24].

## 3. Chronic Gastritis

Based on its topographic localization and aetiological cause(s), chronic gastritis is classified into antrum predominant gastritis (environmental or type B gastritis) and corpus predominant gastritis (autoimmune or type A gastritis) [27].

### 3.1. Antral Predominant Chronic Gastritis

In early work (1862), Crouvelhier [28] observed mucosal inflammation and gastric ulcers in the same stomach. Since then, many studies have been done to identify the pathogenesis of gastric mucosal inflammation and its sequels. Before 1983, ethanol, aspirin, radiation, virus, and fungi were known to induce antral chronic inflammation [29,30]. In the absence of those agents, it was observed that the most common form of gastric mucosal inflammation occurred in the lower social classes and in subjects with blue eyes [29]. However, in 1983, Warren and Marshal [31] discovered *Helicobacter pylori*, the aetiological agent of antral gastritis. Five years afterwards, Correa postulated that the *H. pylori* is the principal agent of the histological events which progress from chronic gastritis to gastric carcinoma through mucosal atrophy, intestinal metaplasia and epithelial dysplasia [32]. At the histological level, antral predominant chronic gastritis ( i.e.infiltrated by lymphocytes, plasma cells and eosinophils) is regarded as active, when infiltrated by neutrophils [33].

Approximately 50% of the world’s population are infected with these bacteria according to some studies [34].

### 3.2. Bacteria in Chronic Gastritis

Years ago, Giannella *et al*. [35] studied, *in vitro*, the bactericidal activity of the normal and achlorhydric gastric juice obtained from various patients. When the pH was less than 4.0, 99.9% of the bacteria were killed within 30 min, *in vitro*, indicating that the gastric bactericidal barrier is primarily pH-hydrochloric-acid dependent, with other constituents of gastric juice contributing little, if any, to the destruction of microorganism [35].

In *H. pylori* infected stomachs, patchy (multifocal) gastric mucosal inflammation is found, first at the *incisura angularis* [36] and subsequently in the antrum and less frequently in the corpus [34,36]. The host reacts to *H. pylori* by increasing the number of T- and B-lymphocytes, followed by infiltration of polymorphonuclear leucocytes. Bacterial adhesion molecules encourage attachment to foveolar cells, while bacteria proteases and ureases damage the gastric epithelium. *H. pylori* infection with strains that possess the cytotoxin-associated gene pathogenicity island secrete a number of bacterial products that cause a severe injury of the gastric mucosa [34,37].

### 3.3. Is Chronic Atrophic Gastritis Specifically Evoked by *H. pylori* and/or by Other Mucosal Insults?

The specific significance of *H. pylori* in the aetiology of chronic gastritis-atrophic gastritis has recently been questioned [38,39,40] as gastric mucosal inflammation might also be caused by factors other than *H. pylori*: viruses, *Candida albicans*, excessive alcohol use, chronic vomiting, retrograde bile reflux, autoantibodies, stress, aspirin, and anti-inflammatory drugs (*v.gr*. NSAID).

Corpus predominant (autoimmune) chronic gastritis is an inflammatory disease triggered by autoantibodies to parietal cells and intrinsic factor [41]. It may lead to the clinical entity, pernicious anemia. The prevalence of pernicious anemia resulting from autoimmune gastritis has been estimated as 127 cases/100,000 inhabitants in Northern Europe including United Kingdom, Denmark, and Sweden [42]. Autoimmune gastritis is characterized by the destruction of the specialized oxyntic glands of the body and fundus by specific lymphocytes. It is restricted to the gastric corpus and fundus, but a total destruction of the oxyntic mucosa only occasionally occurs. Histology reveals periglandular T-lymphocytic (CD3+) infiltration, glandular atrophy, and intestinal metaplasia restricted to the oxyntic mucosa. In advanced forms, the body mucosa is eventually replaced by pseudo-pyloric metaplasia (due to hyperplasia of the mucous neck cell). The mucosa of the antrum remains relatively spared. Autoimmune gastritis is accompanied by a linear or nodular neuroendocrine enterochromaffin-like cell hyperplasia in the corpus [43], or multiple carcinoid tumors.

### 3.4. Bacteria in Corpus Predominant, Autoimmune, Chronic Gastritis

The true role played by *H. pylori* in autoimmune gastritis remains controversial. Due to progressive mucosal atrophy microbial diversity increases with reduced acidity [44,45]. By applying temporal temperature gradient gel electrophoresis and 16S rRNA sequencing, Monstein *et al*. demonstrated particular microbes in the stomach other than *H. pylori*: *Enterococcus*, *Pseudomonas*, *Streptococcus*, *Staphylococcus*, and *Stomatococcus* [45]. Using large-scale 16S rRNA sequencing 128 phylotypes from eight phyla were identified [46], thus, confirming the complexity of microbiota in the gastric mucosa. In patients with antral gastritis, Li *et al*. found 133 phylotypes from eight bacterial phyla as well as 11 *Streptococcus* phylotypes, including *Firmicutes phylum* and *Streptococcus genus* from cultivated biopsies [47]. In the absence of *H. pylori*, other bacterial groups/species seem to be associated with corpus gastritis. Unfortunately, no patients with autoimmune chronic gastritis were included in Li *et al*. [47] studies.

### 3.5. Lysozyme Is Up-Regulated in Chronic Gastritis, Intestinal Metaplasia, Autoimmune Gastritis and Fundic-Gland Polyps

**Figure 5:**
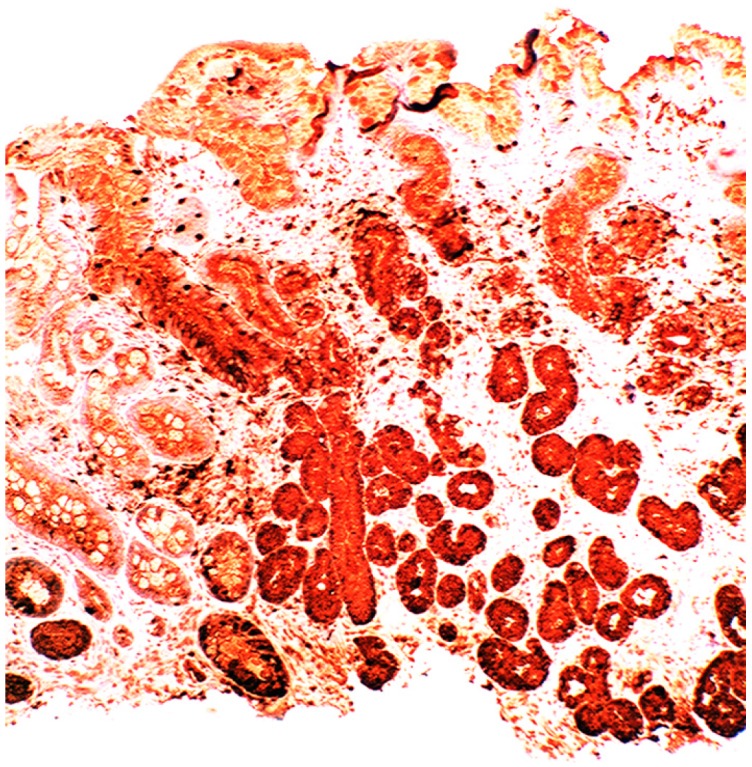
Chronic gastritis (antrum) showing lysozyme expression in surface epithelium, and in antro-pyloric glands (lysozyme immunostain, ×10).

**Figure 6:**
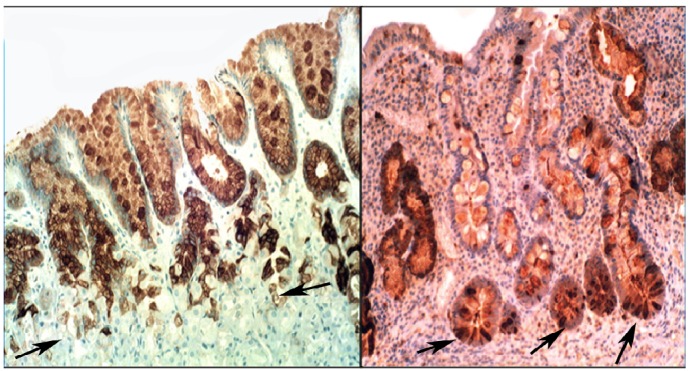
Chronic gastritis with intestinal metaplasia. **Left panel**: Oxyntic (corpus) mucosa showing lysozyme expression in goblet cells and in mucus neck cells. Note absence of lysoyme expression in parietal cells in the lower part of the picture (below arrows); **Right panel**: Antro-pyloric mucosa showing lysozyme expression in goblet cells and in Paneth cells at the bottom of the crypts (arrows, lysozyme immunostain, ×10).

**Figure 7:**
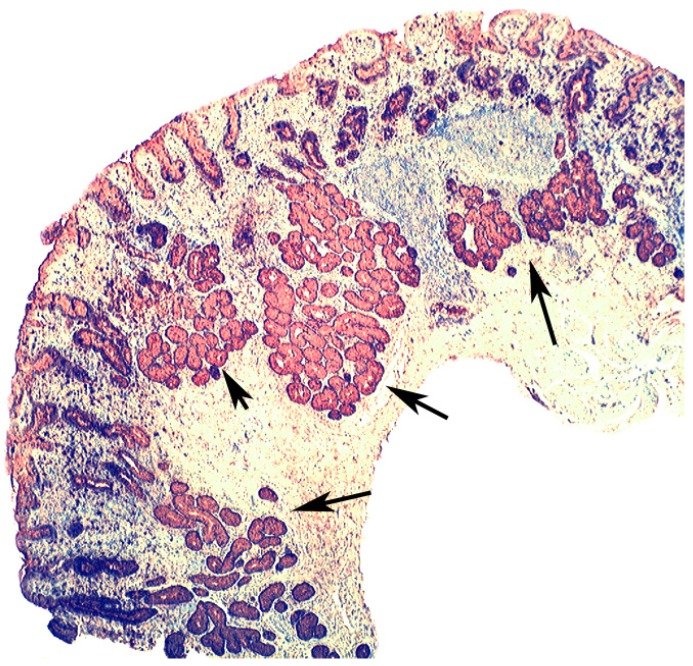
Autoimmune gastritis (corpus) showing lysozyme expression in pseudopyloric glands at arrows (lysozyme immuno-stain, ×4).

In chronic gastritis lysozyme is up-regulated in the neck region of the oxyntic mucosa, in antral pyloric glands (Figure 5) and in the surface-foveolar epithelium of the oxyntic mucosa [8]. In cases with in intestinal metaplasia, lysozyme is up-regulated in goblet cells (Figure 6), and in Paneth cells. In cases with autoimmune gastritis, lysozyme is up-regulated in pseudo-pyloric glands (Figure 7).

The increased lysozyme expression in the goblet cells of gastric intestinal metaplasia appears to be a phenomenon unrelated to the presence of Paneth cells, as lysozyme is similarly over expressed in cases with complete intestinal metaplasia (*i.e.*, having Paneth cells) and with incomplete intestinal metaplasia (*i.e.*, without Paneth cells). As pseudo-pyloric glands are generated by hyperplasia of the mucous neck cells it is not surprising that these cells retain the characteristics of the mucus neck cells, namely to express lysozyme [8]. Human defensin 5, secreted by Paneth cells in the small intestine, may also regulate and maintain microbial balance in the intestinal lumen [48]; in contrast the non-metaplastic atrophic gastric mucosa does not provide a similar defensive reaction. Intestinal metaplasia might evolve following a mucosal insult that affects the stem cells of the crypts of Lieberkhün [26]. Our studies [8,49] suggest that that gastric intestinal metaplasia and gastric atrophy are two different biological processes, atrophy being the result of the local destruction of glands by the chronic inflammation, and intestinal metaplasia, the consequence of an adaptive enzymatic up-regulation aimed to protect the mucosa from proliferating bacteria.

## 4. Intestinal Metaplasia in Disparate Geographical Regions

Years ago, Shousa *et al*. observed a significantly higher prevalence of intestinal metaplasia in gastric biopsies from British patients than in Yemeni patients [50]. In comparative studies of 1675 gastric biopsies and gastrectomy specimens having chronic gastritis we found intestinal metaplasia in 59% of the Japanese patients [51], in 50% of the Italian patients [52], in 32% of the Swedish patients [51], and in 13% of the Mexican patients [52] investigated, suggesting that environmental factors might trigger intestinal metaplasia in chronic gastritis. It has repeatedly been demonstrated that *H. pylori* is absent in areas with intestinal metaplasia or with pseudo-pyloric metaplasia. Hence, it appears safe to postulate that the aim of lysozyme overproduction in intestinal metaplasia and in pseudo-pyloric metaplasia might be to eradicate luminal proliferating bacteria in acid-deficient stomachs.

### 4.1. Fundic Gland Polyps

Fundic gland polyps are small (≤5 mm) nodules of the gastric mucosa characterized by microcysts lined with parietal, chief cells and occasional mucous foveolar cells [53]. Fundic gland polyps are usually found in patients with hereditary diseases, such as familial adenomatous polyposis/Gartner’s syndrome and juvenile polyposis, with non-hereditary (*i.e.*, sporadic) gastric disorders or receiving proton-pump inhibitors. Somatic mutations in the adenomatous polyposis coli (APC) gene are frequent in fundic gland polyps from patients with familial adenomatous polyposis. In sporadic fundic gland polyps other mutations apply such as mutations of the β-catenin gene, a downstream target regulated by the APC protein. However, a common APC/β-catenin pathway seems to be involved in both syndromic and sporadic fundic gland polyps, through the targeting of different genes.

### 4.2. Absence of *H. pylori* in Fundic Gland Polyps

**Figure 8:**
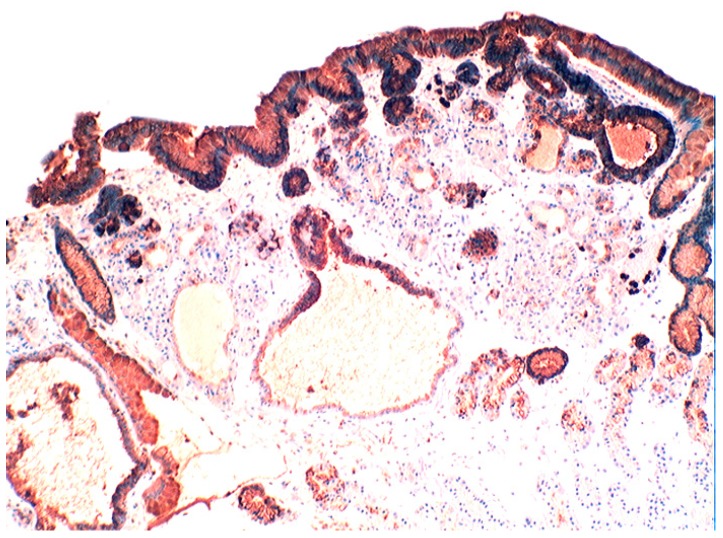
Fundic gland polyp. Marked lyozyme expression in the surface epithelium, in the foveolar pits and in the mucous-producing cells that partly cover the microcysts while the parietal cells were lysozyme negative (lysozyme immunostain, ×10).

Several authors have noticed that *H. pylori* rarely proliferate in the stomach of patients with fundic gland polyps [53]. We also found absence of *H. pylori* infection in a series of fundic gland polyps [54]. The cause(s) for the absence of *H. pylori* infection might be related to lysozyme up-regulation (55]. In fact, lysozyme was found up-regulated in the surface epithelium, in the foveolar pits and in the cells that partly, or entirely, cover dilated glands of fundic gland polyps [55] (Figure 8). The overproduction of lysozyme by the fundic gland polyp epithelium concurred with the absence of *H.* *pylori* in these lesions [55].

### 4.3. Coeliac Disease

Coeliac disease is a common immune-mediated condition in the proximal small intestine, generated by a permanent intolerance to cereal gluten proteins in genetically predisposed individuals [56]. It often leads to mucosal atrophy. In most Western countries, the prevalence of diagnosed coeliac disease in children is 0.5%–1% [57]. In Sweden, the epidemiology of childhood coeliac disease has undergone dramatic changes over recent decade [57]; up to the early 1980s, the incidence of coeliac disease was about 1/1000, but during the mid and late 1980s, a sharp increase was observed at several paediatric clinics. Today, coeliac disease is the second most common chronic disease in Swedish children with an incidence of 3% [58].

Despite numerous sensitive and specific serological markers, duodenal biopsy remains the gold standard for diagnosing coeliac disease. Following diagnosis, a lifelong strict gluten-free diet is the treatment of choice to both restore mucosal normality and revert symptoms and possible complications.

### 4.4. Bacteria in Coeliac Disease

In later years, much attention has been focused on the abnormal microbiota present in the duodenum in patients with coeliac disease. *Bifido bacterium*, *Bacteroides vulgatus*, *Escherichia coli*, and rod-shape bacteria attached to the intestinal epithelium are higher in patients with coeliac disease than in controls whereas *B. bacterium adolescentis*/*B. bacterium animalis lactis* are more prevalent in patients with active coeliac disease than in patients with treated coeliac disease/control patients [59,60,61,62].

### 4.5. Lysozyme is Up-Regulated in Coeliac Disease

**Figure 9:**
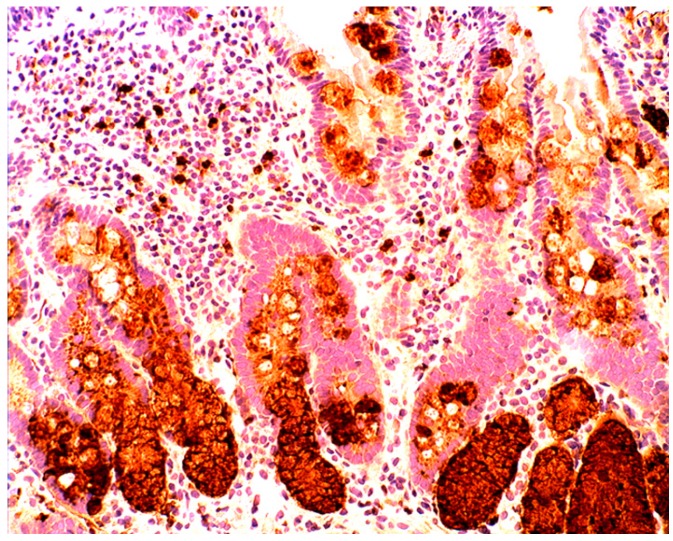
Chronic atrophic duodenitis (celiac disease). Villous atrophy showing lysozyme-rich mucus metaplasia in the lower part of the crypts. Note lysozyme expressing goblet cells (lysozyme immunostain, ×10).

**Figure 10:**
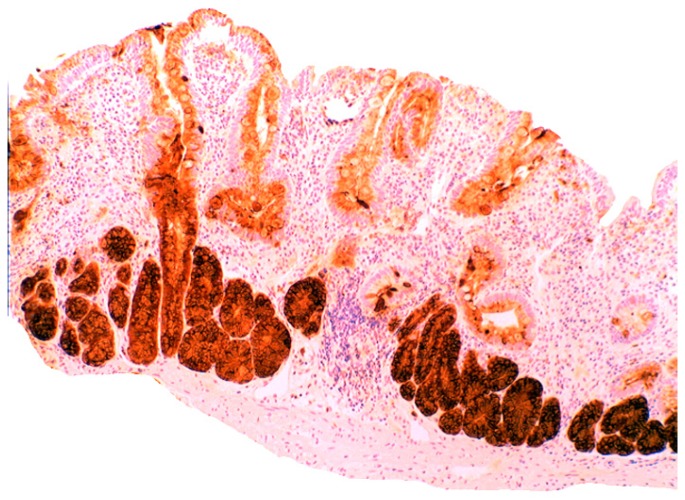
Chronic atrophic duodenitis (celiac disease). Villous atrophy showing extensive lysozyme-rich mucus metaplasia of the crypts. Note absence of Paneth cells (lysozyme immunostain, ×10).

In the normal duodenal mucosa, Paneth cells produce lysozyme. In coeliac disease, lysozyme is up-regulated in goblet cells, in dilated crypts with mucus-metaplasia (Figure 9), a phenomenon more apparent in the *bulbus* [10] (Figure 10). It is not inconceivable that the lysozyme-rich mucus metaplasia mirror stem cell adaptation to the signals generated by the pathogenic bacteria present in the duodenal microenvironment [63].

## 5. Collagenous Colitis, Lymphocytic Colitis, Ulcerative Colitis and Crohn’s Colitis

In 1976, the Swedish pathologist CG Lindström reported the presence of a subepithelial amorphous band in the colonic mucosa in a patient having chronic watery diarrhoea and grossly normal colonoscopy [64]. Lindström called this setting collagenous colitis.

In 1989, Giardelo *et al*. [65] found increased number of intraepithelial lymphocytes in the superficial epithelium of the colon in patients having watery diarrhea and grossly normal colonoscopy, and proposed the term lymphocytic colitis for this type of microscopic colitis.

After an initial rise during the 1980s and early 1990s, the annual incidence of collagenous colitis and lymphocytic colitis has been stable during the past 15 years, in Sweden, at about 5/100,000 inhabitants for each disorder [66].

### 5.1. Bacteria in Microscopic Colitis

Our understanding of a possible altered bacteria flora in microscopic colitis is poor*.* In collagenous colitis Firmicutes and Bacteroidetes were found to dominate the microbiota with seven phylotypes among 50% of the clones: *B. cellulosilyticus*, *B. caccae*, *B. thetaiotaomicron*, *B. uniformis*, *B. dorei*, *B.* spp., and clones showing similarity to *Clostridium clostridioforme* [67]. More recently, Helal *et al*. found an association between *E. coli* and lymphocytic colitis [68].

### 5.2. Lysozyme Is Up-Regulated in Microscopic Colitis

**Figure 11:**
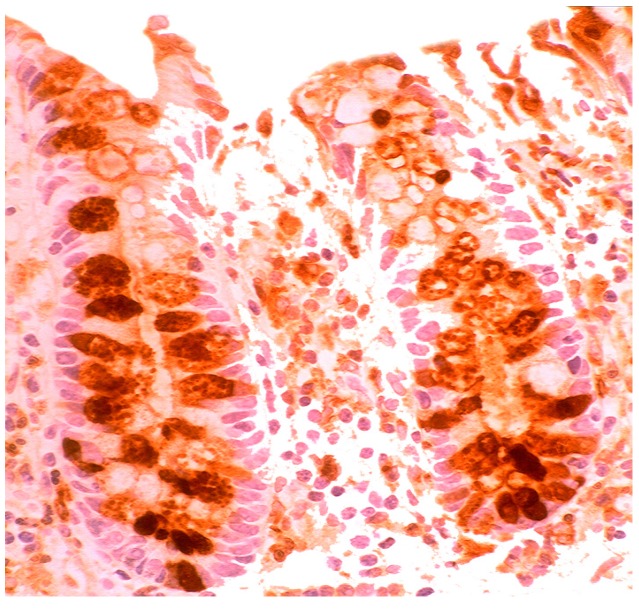
Collagenous colitis. Marked lysozyme immunoreactivity in goblet cells in the crypts (lysozyme immunostain, ×40).

**Figure 12:**
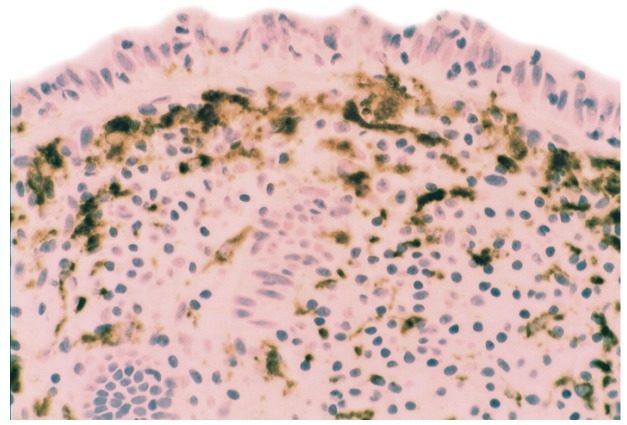
Lymphocytic colitis showing marked lysozyme expression, in macrophages in the *lamina propria* juxtaposing the superficial epithelium (lysozyme immunostain, ×40).

**Figure 13:**
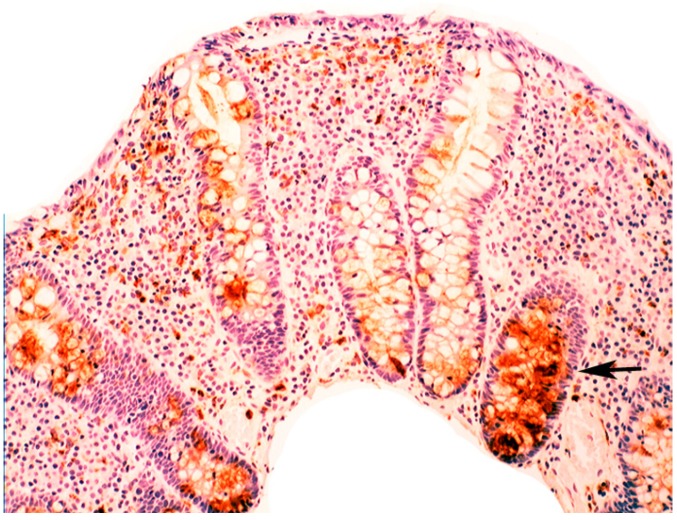
Lymphocyic colitis showing lysozyme expression, in the basal aspect of the crypts (arrow, lysozyme immunostain, ×20).

In collagenous colitis lysozyme is up-regulated in the colonic crypts and in metaplastic Paneth cells [9] (Figure 11). In lymphocytic colitis, lysozyme is up-regulated in macrophages underlying the surface epithelium of the *lamina propria* [9] (Figure 12), as well as in the lower part of the crypts (Figure 13).

The increased production of lysozyme in collagenous colitis and in lymphocytic colitis supports a bacterial aetiology for these two diseases. The different mucosal cell types displaying increased production of lysozyme (epithelial *vs*. macrophages) substantiates the notion that collagenous colitis and lymphocytic colitis might be two different diseases.

Notably, collagenous colitis and lymphocytic colitis are also found in non-human primates having protracted intractable diarrhoea [69].

### 5.3. Bacteria in Inflammatory Bowel Disease

Our understanding of a possible altered bacteria flora in inflammatory bowel disease is less clear. In ulcerative colitis, bacterial diversity is reduced, including Clostridium groups IV, XIVa (*Faecalibacterium prausnitzii*) and Bifidobacteria and lactobacillia. On the other hand, *C. difficile* is increased [70,71,72,73,74,75,76,77,78,79,80,81].

In Crohn’s colitis, the number of mucosal bacteria such as *Mycobacterium avium paratuberculosis*, *C. difficile*, *Ruminococcus gnavus*, *Enterobacteriaceae*, and *E. Coli* are increased, while bacterial diversity, Clostridium groups IV, XIVa (*F. prausnitzii*), and Bifidobacteria and Lactobacillia, are reduced [76,77,78,79].

### 5.4. Lysozyme Up-Regulated in Inflammatory Bowel Disease

**Figure 14:**
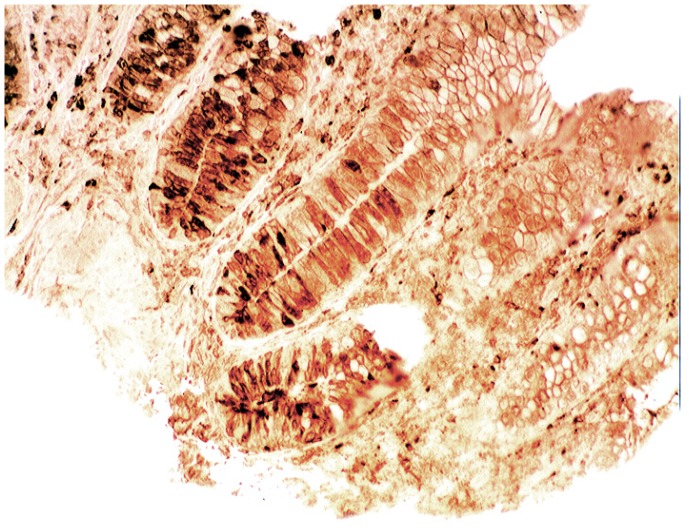
Ulcerative colitis (active phase). Marked lysozyme immunoreactivity in the goblet cells in the crypts (lysozyme immunostain, ×10).

**Figure 15:**
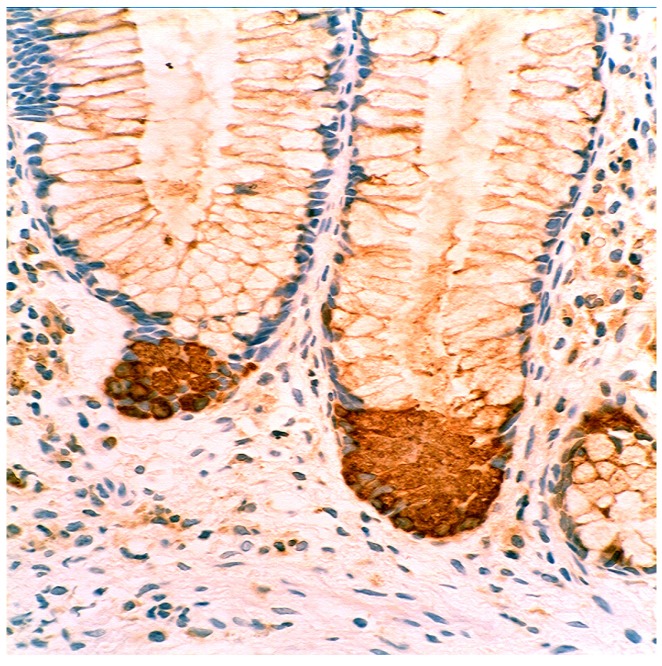
Ulcerative colitis (in remission). Marked lysozyme immunoreactivity in hyperplastic- metaplastic Paneth cells in the bottom of the crypts of the left colon, (lysozyme immunostain, ×10).

**Figure 16:**
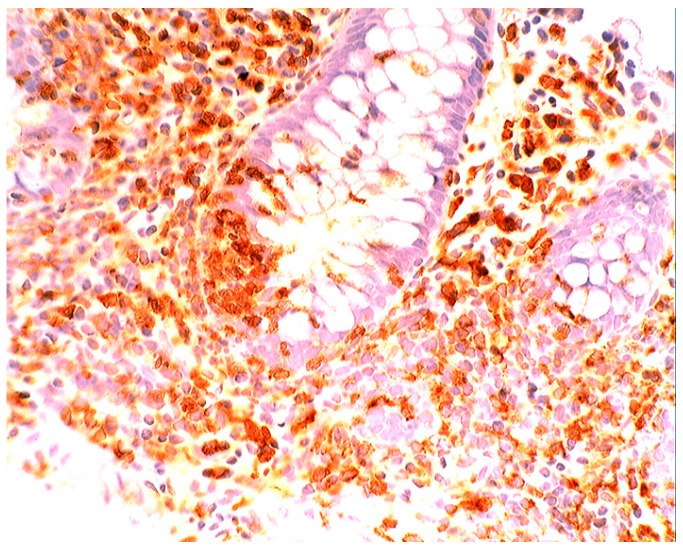
Crohn colitis: Marked lysozyme immunoreactivity in macrophages in the *lamina propria* and in epithelial cells at the bottom of the crypts (lysozyme immunostain, ×20).

In active ulcerative colitis, lysozyme is up-regulated in metaplastic Paneth cells (left colon) and in the deep half of the crypts (Figure 14). In ulcerative colitis in remission, lysozyme is up-regulated in metaplastic Paneth cells [9] (left colon, Figure 15). No lysozyme expression is recorded in the crypts. In Crohn’s colitis lysozyme up-regulation is found in metaplastic Paneth cells (left colon), in the crypts as well as in the *lamin propria mucosae* [9] (Figure 16). The increased lysozyme production in the colonic mucosa in patients with inflammatory bowel disease may highlight an amplified mucosal protection against the pathogenic bacteria proliferating in the colonic microenvironment in these patients [70,71,72,73,74,75,76,77,78,79,80,81].

## 6. Epilogue

Today, bacterial antibiotic resistance continues to exhaust our supply of effective antibiotics. The future challenge is how to solve the conundrum of bacterial resistance to antibacterial drugs. In his Presidential address, Alexander Fleming said, 80 years ago: “I choose lysozyme as the subject for this address for two reasons, firstly because I have a fatherly interest in the name and, secondly, because its importance in connection with natural immunity does not seem to be generally appreciated” [3]. Perhaps, the challenging legacy of Alexander Fleming, together with the more recent bacterial resistance to commercial antibiotics, offer the explanation for the boost in research on the potential use of lysozyme for the treatment of infectious diseases. This research includes not only laboratory and farmed animals, but also agricultural products [82,83,84,85,86,87,88,89,90,91].

## References

[1] Kaper J.B., Sperandio V. (2005). Bacterial cell-to-cell signaling in the gastrointestinal tract. Infect. Immun..

[2] Dominguez-Bello M.G., Blaser M.J., Ley R.E., Knight R. (2011). Development of the human gastrointestinal microbiota and insights from high-throughput sequencing. Gastroenterology.

[3] Fleming A. (1922). On a remarkable bacteriolytic element found in tissues and secretions. Proc. R. Soc. Sect. B.

[4] Yoshimura K., Toibana A., Nakahama K. (1988). Human lysozyme: Sequencing of a cDNA, and expression and secretion by *Saccharomyces cerevisiae*. Biochem. Biophys. Res. Commun..

[5] Peters C., Kruse U., Pollwein R., Grzeschik K., Sippel T. (1989). The human lysozyme gene. Sequence organization and chromosomal localization. Eur. J. Biochem..

[6] Sahoo N.R., Kumar P., Bhusan B., Bhattacharya T.K., Dayal S., Sahoo M. (2012). Lysozyme in livestock: A guide to selection for disease resistance: A review. J. Anim. Sci. Adv..

[7] Rubio C.A., Lörinc E. (2011). Lysozyme is up-regulated in Barrett’s mucosa. Histopathology.

[8] Rubio C.A., Befrits R. (2009). Increased lysozyme expression in gastric biopsies with intestinal metaplasia and pseudopyloric metaplasia. Int. J. Clin. Exp. Med..

[9] Rubio C.A. (2011). Lysozyme expression in microscopic colitis. J. Clin. Pathol..

[10] Rubio C.A. (2011). Lysozyme-rich mucus metaplasia in duodenal crypts supersedes Paneth cells in celiac disease. Virchows Arch..

[11] Appelman H.D., Umar A., Orlando R.C., Sontag S.J., Nandurkar S., el-Zimaity H., Lanas A., Parise P., Lambert R., Shields H.M. (2011). Barrett’s esophagus: Natural history. Ann. N. Y. Acad. Sci..

[12] Rubio C.A., Dick E.J., Schlabritz-Loutsevitch N.E., Orrego A., Hubbard G.B. (2009). The columnar-lined mucosa at the gastroesophageal junction in non-human primates. Int. J. Clin. Exp. Pathol..

[13] Rubio C.A., Nilsson J.R., Owston M., Dick E.J. (2012). The length of the Barrett's mucosa in baboons, revisited. Anticancer Res..

[14] Spechler S., Goyal R.K. (1986). Barrett’s esophagus. N. Engl. J. Med..

[15] Sampliner R.E. (1998). Practice guidelines on the diagnosis, surveillance, and therapy of Barrett’s esophagus. The Practice Parameters Committee of the American College of Gastroenterology. Am. J. Gastroenterol..

[16] Playford R.J. (2006). New British Society of Gastroenterology (BSG) guidelines for the diagnosis and management of Barrett’s oesophagus. Gut.

[17] Fiocca R., Mastracci L., Milione M., Parente P., Savarino V. (2011). Gruppo Italiano Patologi Apparato Digerente (GIPAD) and Società Italiana di Anatomia Patologica e Citopatologia Diagnostica/International Academy of Pathology, Italian division (SIAPEC/IAP). Microscopic esophagitis and Barrett’s esophagus: The histology report. Dig. Liver Dis..

[18] Takubo K., Vieth M., Aida J., Sawabe M., Kumagai Y., Hoshihara Y., Arai T. (2009). Differences in the definitions used for esophageal and gastric diseases in different countries: Endoscopic definition of the esophagogastric junction, the precursor of Barrett’s adenocarcinoma, the definition of Barrett’s esophagus, and histologic criteria for mucosal adenocarcinoma or high-grade dysplasia. Digestion.

[19] Waldum H.L., Hauso Ø., Sandvik A.K. (2010). PPI-induced hypergastrinaemia and Barrett’s mucosa: The fog thickens. Gut.

[20] Yang L., Lu X., Nossa C.W., Francois F., Peek R.M., Pei Z., Yang L., Lu X., Nossa C.W., Francois F. (2009). Inflammation and intestinal metaplasia of the distal esophagus are associated with alterations in the microbiome. Gastroenterology.

[21] Pei Z., Yang L., Peek R.M., Levine S.M., Pride D.T., Blaser M.J. (2005). Bacterial biota in reflux esophagitis and Barrett’s esophagus. World J. Gastroenterol..

[22] Macfarlane S., Furrie E., Macfarlane G., Dillon J. (2007). Microbial colonization of the upper gastrointestinal tract in patients with Barrett’s esophagus. Clin. Infect. Dis..

[23] Liu N., Ando T., Ishiguro K., Maeda O., Watanabe O., Funasaka K., Nakamura M., Miyahara R., Ohmiya N., Goto H. (2013). Characterization of bacterial biota in the distal esophagus of Japanese patients with reflux esophagitis and Barrett’s esophagus. BMC Infect. Dis..

[24] Rubio C.A. (2012). Lysozyme is up-regulated in columnar-lined Barrett’s mucosa: A possible natural defence mechanism against Barrett’s esophagus-associated pathogenic bacteria. Anticancer Res..

[25] Sarosi G., Brown G., Jaiswal K., Feagins L.A., Lee E., Crook T.W., Souza R.F., Zou Y.S., Shay J.W., Spechler S.J. (2008). Bone marrow progenitor cells contribute to esophageal regeneration and metaplasia in a rat model of Barrett’s esophagus. Dis. Esophagus.

[26] Rubio C.A., Singh S.R. (2011). Putative Stem Cells in Mucosas of the Esophago-Gastrointestinal Tract. Stem Cell, Regenerative Medicine and Cancer.

[27] Misiewicz J., Tygat G., Goodwin C. (1990). The Sydney System: A new classification of gastritis. Work. Party Rep..

[28] Cruveihier J., Bulletins de la Société d'anthropologie de Paris (1862). Traté d´anatomie pathologique genéràle.

[29] MacDonald W., Rubin C. (1967). Gastric biopsy: A critical evaluation. Gastroenterology.

[30] Edwards F., Coghill N. (1966). Aetiological factors in chronic atrophic gastritis. Br. Med. J..

[31] Warren J., Marshall B. (1983). Unidentified curved bacilli on gastric epithelium in active chronic gastritis. Lancet.

[32] Correa P. (1988). A human model of gastric carcinogenesis. Cancer Res..

[33] Oh J.D., Kling-Backhed H., Giannakis M., Engstrand L.G., Gordon J.I. (2006). Interactions between gastric epithelial stem cells and *Helicobacter pylori* in the setting of chronic atrophic gastritis. Curr. Opin. Microbiol..

[34] Dixon M.F., Genta R.M., Yardley J.H., Correa P. (1996). Classification and grading of gastritis: The updated Sydney System. International workshop on the histopathology of gastritis, Houston 1994. Am. J. Surg. Pathol..

[35] Giannella R., Broitman S., Zamcheck N. (1972). Gastric acid barrier to ingested micro-organisms in man: Studies *in vivo* and in *vitro*. Gut.

[36] Rubio C.A., Jaramillo E., Suzuki G., Lagergren P., Nesi G. (2009). Antralization of the gastric mucosa of the incisura angularis an its gastrin expression. Int. J. Clin. Exp. Pathol..

[37] Nakajima S., Nishiyama Y., Yamaoka M., Yasuoka T., Cho E. (2010). Changes in the prevalence of *Helicobacter pylori* infection and gastrointestinal diseases in the past 17 years. J. Gastroenterol. Hepatol..

[38] Campbell D., Warren B., Thomas J., Figura N., Telford J., Sullivan P. (2001). The African enigma: Low prevalence of gastric atrophy, high prevalence of chronic inflammation in West African adults and children. Helicobacter.

[39] Graham D.Y., Lu H., Yamaoka Y. (2009). African, Asian or Indian enigma, the East Asian *Helicobacter pylori*: Facts or medical myths. J. Dig. Dis..

[40] Saieva C., Rubio C.A., Nesi G., Zini E., Filomena A. (2012). Classification of gastritis in first-degree relatives of patients with gastric cancer in a high cancer-risk area in Italy. Anticancer Res..

[41] Torbenson M., Abraham S.C., Boitnott J., Yardley J.H., Wu T. (2002). Autoimmune gastritis: Distinct histological and immuno-histochemical findings before complete loss of oxyntic glands. Mod. Pathol..

[42] Petersson F., Borch K., Franzén E. (2002). Prevalence of subtypes of intestinal metaplasia in the general population and in patients with autoimmune chronic atrophic gastritis. Scand. J. Gastroenterol..

[43] Rubio C.A., Kaufeldt A. (2013). Paucity of synaptophysin-expressing cells in Barrett’s mucosa. Histopathology.

[44] Guerre J., Vedel G., Gaudric M., Paul G., Cornuau J. (1986). Bacterial flora in gastric juice taken at endoscopy in 93 normal subjects. Pathol. Biol..

[45] Monstein H.J., Tiveljung A., Kraft C.H., Borch K., Jonasson J. (2000). Profiling of bacterial flora in gastric biopsies from patients with *Helicobacter pylori* associated gastritis and histologically normal control individuals by temperature gradient gel electrophoresis and 16S rDNA sequence analysis. J. Med. Microbiol..

[46] Bik E.M., Eckburg P.B., Gill S.R., Nelson K.E., Purdom E.A. (2006). Molecular analysis of the bacterial microbiota in the human stomach. Proc. Natl. Acad. Sci. USA.

[47] Li X.X., Wong G.L., To K.F., Wong V.W., Lai L.H. (2009). Bacterial microbiota profiling in gastritis without *Helicobacter pylori* infection or non-steroidal anti-Inflammatory drug use. PLoS ONE.

[48] Shen B., Porter E.M., Reynoso E., Shen C., Ghosh D., Connor J.T., Drazba J., Rho H.K., Gramlich T.L., Li R. (2005). Human defensin 5 expression in intestinal metaplasia of the upper gastrointestinal tract. J. Clin. Pathol..

[49] Rubio C.A. (2007). My approach to reporting a gastric biopsy. J. Clin. Pathol..

[50] Shousha S., el-Sherif A.M., el-Guneid A., Arnaout A.H. (1993). *Helicobacter pylori* and intestinal metaplasia: Comparison between British and Yemeni patients. Am. J. Gastroenterol..

[51] Rubio C.A., Kato Y., Sugano H., Kitagawa T. (1987). Intestinal metaplasia of the stomach in Swedish and Japanese patients without ulcers or carcinoma. Jpn. J. Cancer Res..

[52] Rubio C.A., Jessurum J., Kato Y. (1992). Low frequency of intestinal metaplasia in gastric biopsies from Mexican patients: A comparison with Japanese and Swedish patients. Jpn. J. Cancer Res..

[53] Shand A., Taylor A., Banerjee M., Lessels A., Coia J., Clark C., Haites N., Ghosh S. (2002). Gastric fundic gland polyps in south-east Scotland: Absence of adenomatous polyposis coli gene mutations and a strikingly low prevalence of Helicobacter pylori infection. J. Gastroenterol. Hepatol..

[54] Rubio C.A. (2009). Plugs clog the glandular outlets in fundic gland polyps. Int. J. Clin. Exp. Pathol..

[55] Rubio C.A. (2010). Lysozyme overexpression in fundic gland polyps. Anticancer Res..

[56] Dewar D.H., Ciclitira P.J. (2005). Clinical features and diagnosis of celiac disease. Gastroenterology.

[57] Ivarsson A., Högberg L., Stenhammar L., Swedish Childhood Coeliac Disease Working Group (2010). The swedish childhood coeliac disease working group after 20 years: History and future. Acta Paediatr..

[58] Myléus A., Ivarsson A., Webb C., Danielsson L., Hernell O., Högberg L., Karlsson E., Lagerqvist C., Norström F., Rosén A. (2009). Celiac disease revealed in 3% of Swedish 12-year-olds born during an epidemic. J. Pediatr. Gastroenterol. Nutr..

[59] Sánchez E., Donat E., Ribes-Koninckx C., Calabuig M., Sanz Y. (2010). Intestinal *Bacteroides* species associated with coeliac disease. J. Clin. Pathol..

[60] Schippa S., Lebba V., Barbato M., di Nardo G., Totino V., Checchi M.P., Longhi C., Maiella G., Cucchiara S., Conte M.P. (2010). A distinctive “microbial signature” in celiac pediatric patients. BMC Microbiol..

[61] Forsberg G., Fahlgren A., Hörstedt P., Hammarström S., Hernell O., Hammarström M.L. (2004). Presence of bacteria and innate immunity of intestinal epithelium in childhood celiac disease. Am. J. Gastroenterol..

[62] Ou G., Hedberg M., Hörstedt P., Baranov V., Forsberg G., Drobni M., Sandström O., Hammarström S. (2009). Proximal small intestinal microbiota and identification of rod-shaped bacteria associated with childhood celiac disease. Am. J. Gastroenterol..

[63] Rubio C.A., Singh S.R. (2013). Signaling pathways, gene regulation and duodenal neoplasias. Signaling, Gene Regulation and Cancer.

[64] Lindström C.G. (1976). “Collagenous colitis” with watery diarrhoea: A new entity?. Pathol Eur..

[65] Giardiello F.M., Lazenby A.J., Bayless T.M., Levine E.J., Bias W.B., Ladenson P.W., Yardley J.H. (1989). Lymphocytic (microscopic) colitis. Clinico-pathologic study of 18 patients and comparison to collagenous colitis. Dig. Dis. Sci..

[66] Wickbom A., Bohr J., Eriksson S., Udumyan R., Nyhlin N., Tysk C. (2013). Stable incidence of collagenous colitis and lymphocytic colitis in Örebro, Sweden, 1999–2008: A continuous epidemiologic study. Inflamm. Bowel Dis..

[67] Gustafsson R.J., Ohlsson B., Benoni C., Jeppsson B., Olsson C. (2012). Mucosa-associated bacteria in two middle-aged women diagnosed with collagenous colitis. World J. Gastroenterol..

[68] Helal T.E., Ahmed N.S., el Fotoh O.A. (2005). Lymphocytic colitis: A clue to bacterial etiology. World J. Gastroenterol..

[69] Rubio C.A., Hubbard G.B. (2001). Chronic colitis in baboons: Similarities with chronic colitis in humans. In Vivo.

[70] Khalil N.A., Walton G.E., Gibson G.R., Tuohy K.M., Andrews S.C. (2014). *In vitro* batch cultures of gut microbiota from healthy and ulcerative colitis (UC) subjects suggest that sulphate-reducing bacteria levels are raised in UC and by a protein-rich diet. Int. J. Food Sci. Nutr..

[71] Kumari R., Ahuja V., Paul J. (2013). Colonisation by Faecalibacterium prausnitzii and maintenance of clinical remission in patients with ulcerative colitis. World J. Gastroenterol..

[72] Varela E., Manichanh C., Gallart M., Torrejón A., Borruel N., Casellas F., Guarner F., Antolin M. (2013). Colonisation by *Faecalibacterium prausnitzii* and maintenance of clinical remission in patients with ulcerative colitis. Aliment. Pharmacol. Ther..

[73] Pilarczyk-Zurek M., Chmielarczyk A., Gosiewski T., Tomusiak A., Adamski P., Zwolinska-Wcislo M., Mach T., Heczko P., Strus M. (2013). Possible role of Escherichia coli in propagation and perpetuation of chronic inflammation in ulcerative colitis. BMC Gastroenterol..

[74] Yukawa T., Ohkusa T., Shibuya T., Tsukinaga S., Mitobe J., Takakura K., Takahara A., Odahara S., Tajiri H. (2013). Nested culture method improves detection of Fusobacterium from stool in patients with ulcerative colitis. Jpn. J. Infect. Dis..

[75] Kabeerdoss J., Sankaran V., Pugazhendhi S., Ramakrishna B.S. (2013). Clostridium leptum group bacteria abundance and diversity in the fecal microbiota of patients with inflammatory bowel disease: A case-control study in India. BMC Gastroenterol..

[76] Gałecka M., Szachta P., Bartnicka A., Łykowska-Szuber L., Eder P., Schwiertz A. (2013). Faecalibacterium prausnitzii and Crohn’s disease—Is there any connection?. Pol. J. Microbiol..

[77] Kale-Pradhan P.B., Zhao J., Palmer J., Wilhelm S.M. (2013). The role of antimicrobials in Crohn’s disease. Expert. Rev. Gastroenterol. Hepatol..

[78] Nickerson K., McDonald C. (2012). Crohn’s disease-associated adherent-invasive Escherichia coli adhesion is enhanced by exposure to the ubiquitous dietary polysaccharide maltodextrin. PLoS One.

[79] Erickson A., Cantarel B., Lamendella R., Darzi Y., Mongodin E., Pan C., Shah M., Halfvarson J., Jansson J.K. (2012). Integrated metagenomics/metaproteomics reveals human host-microbiota signatures of Crohn’s disease. PLoS One.

[80] Jostins L., Ripke S., Weersma R.K., Duerr R.H., McGovern D.P., Hui K.Y., Lee J.C., Schumm L., Sharma Y., Anderson C. (2012). Host-microbe interactions have shaped the genetic architecture of inflammatory bowel disease. Nature.

[81] Shanahan F. (2012). The microbiota in inflammatory bowel disease: Friend, bystander, and sometime-villain. Nutr. Rev..

[82] Muriel-Galet V., Talbert J.N., Hernandez-Munoz P., Gavara R., Goddard J.M. (2013). Covalent immobilization of lysozyme on ethylene vinyl alcohol films for nonmigrating antimicrobial packaging applications. J. Agric. Food Chem..

[83] Ibrahim H.R., Imazato K., Ono H. (2011). Human lysozyme possesses novel antimicrobial peptides within its *N*-terminal domain that target bacterial respiration. J. Agric. Food Chem..

[84] Oliver W.T., Wells J.E. (2013). Lysozyme as an alternative to antibiotics improves growth performance and small intestinal morphology in nursery pigs. J. Anim. Sci..

[85] Teneback C., Scanlon T., Wargo M., Bement J., Griswold K., Leclair L.W. (2013). Bioengineered lysozyme reduces bacterial burden and inflammation in a murine model of mucoid Pseudomonas aeruginosa lung infection. Antimicrob. Agents Chemother..

[86] Pridgeon J.W., Klesius P.H., Dominowski P.J., Yancey R.J., Kievit M.S. (2013). Chicken-type lysozyme in channel catfish: Expression analysis, lysozyme activity, and efficacy as immunostimulant against *Aeromonas hydrophila* infection. Fish Shellfish Immunol..

[87] Cegielska-Radziejewska R., Szablewski T. (2013). Effect of modified lysozyme on the microflora and sensory attributes of ground pork. J. Food Prot..

[88] Lamppa J.W., Tanyos S.A., Griswold K.E. (2013). Engineering *Escherichia coli* for soluble expression and single step purification of active human lysozyme. J. Biotechnol..

[89] Rosu V., Bandino E., Cossu A. (2013). Unraveling the transcriptional regulatory networks associated to mycobacterial cell wall defective form induction by glycine and lysozyme treatment. Microbiol. Res..

[90] Scanlon T.C., Teneback C.C., Gill A., Bement J.L., Weiner J.A., Lamppa J., Leclair L.W., Griswold K.E. (2010). Enhanced antimicrobial activity of engineered human lysozyme. ACS Chem. Biol..

[91] Bhavsar T., Liu M., Hardej D., Liu X., Cantor J. (2010). Aerosolized recombinant human lysozyme ameliorates Pseudomonas aeruginosa-induced pneumonia in hamsters. ACS Chem. Biol..

